# Knowledge and Attitude of Parents Regarding the Human Papillomavirus Vaccine as a New Component in the Saudi Vaccination Schedule

**DOI:** 10.7759/cureus.52508

**Published:** 2024-01-18

**Authors:** Abdulrahman A Bin Alamir, Abdullah H Almotairi, Fahad H Almutairi, Khaled N Almujel, Abdalah E Almhmd, Abdulrahman F Alanazi, Khalid F Alotaibi, Mohammed H Alanazi, Amer S Alsaeri, Meshal A Alruwaili, Rayan S Alghamdi

**Affiliations:** 1 Department of Pediatrics, College of Medicine, Majmaah University, Majmaah, SAU; 2 College of Medicine, Majmaah University, Majmaah, SAU; 3 General Practice, Health Home Care, Aseer Central Hospital, Abha, SAU

**Keywords:** hpv vaccine, knowledge, attitude, saudi parents, human papillomavirus (hpv)

## Abstract

Background

The most frequent gynecologic cancer in women is cervical cancer. The majority of incidents take place in less developed nations without access to reliable screening tools. Human papillomavirus (HPV) exposure, smoking, and immune system dysfunction are risk factors. As a result of effective screening, its incidence and death have significantly decreased in many nations. Hence, this study aims to assess the level of knowledge and awareness among parents regarding HPV, including its associated health risks and the benefits of vaccination.

Methodology

A descriptive cross-sectional study was carried out in the Riyadh region of Saudi Arabia from September to November 2023. The main tool used for gathering data was an online, self-administered survey via Google Forms. Collected data was analysed using SPSS v. 24 (IBM Corp., Armonk, NY), where all applicable statistical tests were used.

Results

Females exhibited higher levels of confidence and agreement with COVID-19 and HPV vaccination recommendations compared to males. A substantial percentage of males expressed strong disagreement and reduced confidence in HPV vaccination, contributing to the gender-based divergence. Individuals with higher education levels, such as university degree graduates, showed greater support for compulsory vaccines and a preference for natural immunity development in their children. Marital status played a role in vaccine-related decisions, with variations in vaccine refusal rates and difficulty discussing the HPV vaccine noted among individuals based on their marital status.

Conclusion

The study highlights the value of medical experts and specifically created training programs to close knowledge gaps and boost HPV vaccination rates. Demographic factors have an impact on attitudes, which highlights the need for targeted interventions.

## Introduction

Cervical cancer is a significant health concern globally, ranking as the fourth most common cancer and the fourth leading cause of cancer death among women [1,2]. In Saudi Arabia, it is the 13th most common malignancy in women. Human papillomavirus (HPV) is the most common cause of cervical cancer among Saudi females of reproductive age [3]. Therefore, it is crucial to increase awareness and knowledge among parents about the benefits of HPV vaccination for young females to prevent the development of cervical cancer [4]. Parents' attitudes and knowledge of HPV vaccination may affect their adherence to vaccinating their daughters, emphasizing the importance of education and outreach efforts [1]. Human papillomavirus (HPV) is a significant risk factor for cervical cancer, with HPV types 16 and 18 being responsible for more than 70% of all cervical cancers in females and genital and oropharyngeal cancers in both males and females [5, 6]. HPV is the most common sexually transmitted infection and can be transmitted through skin-to-skin contact. However, the HPV vaccine is proven to prevent infection and reduce the incidence of cervical cancer in females and anogenital cancers in both genders [7]. In Saudi Arabia, the HPV vaccine is estimated to protect against two-thirds of cervical cancer cases, highlighting the importance of vaccination programs to prevent the spread of the virus and reduce the incidence of associated cancers [8]. It is important to note that there is currently no virus-specific treatment for HPV infection. Therefore, prevention through vaccination is crucial for reducing the incidence of HPV-related diseases, including cervical cancer. In addition to vaccination efforts, it is necessary to increase awareness and knowledge about HPV infection in the general population to improve management and treatment options. Understanding attitudes and knowledge about HPV can help healthcare providers develop effective prevention and treatment strategies and improve patient outcomes. Overall, a comprehensive approach that includes vaccination, education, and screening is necessary to manage and reduce the impact of HPV infection effectively [9].

The general objective of this study is to assess the level of knowledge and awareness among parents regarding HPV, including its associated health risks and the benefits of vaccination. To achieve this overarching goal, specific objectives have been outlined. Firstly, the study aims to identify the factors that influence parents' attitudes toward HPV vaccination. Additionally, it seeks to determine the willingness of parents to vaccinate their children against HPV and explore their preferences regarding vaccine administration [10]. Furthermore, the study aims to evaluate the effectiveness of educational interventions in enhancing parents' knowledge and awareness of HPV, ultimately promoting the uptake of HPV vaccination. In addressing potential obstacles, the research aims to identify barriers to HPV vaccination uptake among parents and develop strategies to overcome these challenges. Lastly, the study aims to investigate the impact of social and cultural norms on parents' decisions regarding the vaccination of their children against HPV. Through these specific objectives, the research endeavours to provide a comprehensive understanding of factors influencing parental attitudes and behaviours related to HPV vaccination.

## Materials and methods

Study design

This study adopted a descriptive cross-sectional design to assess the level of knowledge and awareness among parents regarding HPV, including its associated health risks and the benefits of vaccination.

Study setting

This is a community-based study and all parents who fulfilled inclusion criteria and agreed to participate in this study were selected.

Sample size

A total of 281 participants who agreed to be part of this study were selected. The inclusion criteria for this study include parents with children aged 1-14 who live in the Riyadh region. The exclusion criteria were parents with children above 14 years old. Additionally, parents who do not live in the Riyadh region were excluded because this study aims to examine parents' experiences within this geographical context.

Research instrument

In the present study, a questionnaire was used for data collection. A questionnaire is a data collection method completed by a respondent in a written format. The questionnaire was divided into two parts: Part 1 covered demographic characteristics, and Part 2 covered variables related to knowledge and awareness of parents regarding to HPV and its associated risk factors and the benefits of vaccination.

Data collection

The recruitment process began in September: a Google Form was shared by sending the link to the study population through social media platforms including Facebook, WhatsApp, Telegram, and Twitter.

Data analysis

After collection, the data were analysed using SPSS v. 24 for Windows (IBM Corp., Armonk, NY) and all descriptive and analytical statistical tests were applied.

Ethical considerations

All ethical standards for conducting this study were adhered to. Ethical approval was granted by the Majmaah University for Research Ethics committee on 17 September 2023 with approval number MUREC, Sep.17/COM-2023 /28-2.

## Results

**Figure 1:**
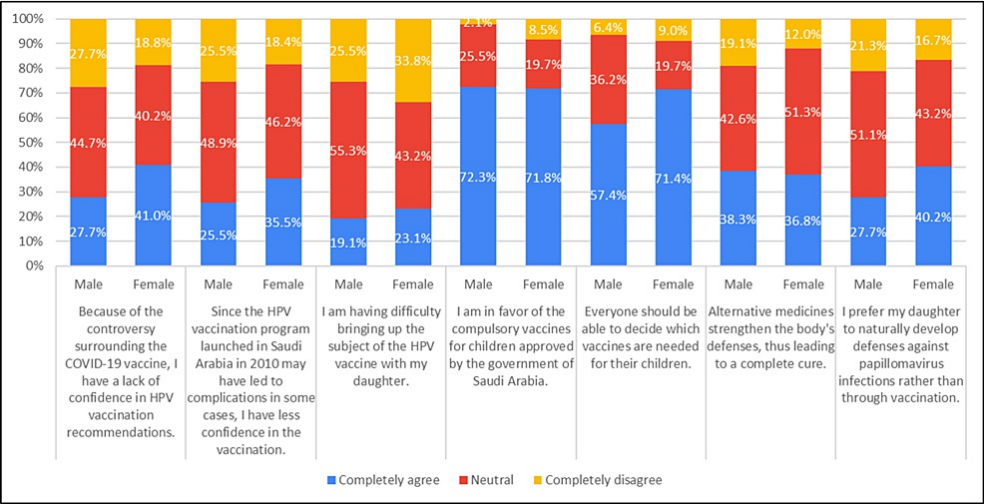
Association of attitudes with gender (N=281)

Approximately 41% of females express complete agreement, while 27.7% of males strongly disagree with the controversy surrounding the COVID-19 vaccine, showing reduced confidence in HPV vaccination recommendations. About 35.5% of females completely agree, contrasting with 25.5% of males who entirely disagree, attributing their decreased confidence in vaccination to potential complications. Regarding discussions about the HPV vaccine, around 23.1% of females fully agree, while 25.5% of males completely disagree, citing difficulty in broaching the subject with their daughters. In support of compulsory vaccines for children approved by the Saudi Arabian government, 72.3% of males completely agree, whereas 8.5% of females express complete disagreement. When it comes to vaccine decision-making, 71.4% of females fully agree, while 9.0% completely disagree, asserting their right to determine the vaccines necessary for their children. In the context of alternative medicines, 36.8% of females entirely agree, but 12.0% of females strongly disagree with the notion that such remedies enhance the body's defense, leading to a complete cure. Finally, 40.2% of females are in complete agreement, in contrast to 21.3% of males who completely disagree, expressing a preference for their daughters to naturally develop defenses against papillomavirus infections rather than through vaccination (Table 1, Figure 1).

**Table 1:** Association of attitudes with gender (N=281)

Attitude	Sex	Completely agree	Neutral	Completely disagree	Chi-square, P-value
Because of the controversy surrounding the COVID-19 vaccine, I have a lack of confidence in HPV vaccination recommendations.	Male	13 (27.7%)	21 (44.7%)	13 (27.7%)	3.510, 0.173
	Female	96 (41.0%)	94 (40.2%)	44 (18.8%)	
Since the HPV vaccination program launched in Saudi Arabia in 2010 may have led to complications in some cases, I have less confidence in the vaccination.	Male	12 (25.5%)	23 (48.9%)	12 (25.5%)	2.232, 0.328
	Female	83 (35.5%)	108 (46.2%)	43 (18.4%)	
I am having difficulty bringing up the subject of the HPV vaccine with my daughter.	Male	9 (19.1%)	26 (55.3%)	12 (25.5%)	2.368, 0.306
	Female	54 (23.1%)	101 (43.2%)	79 (33.8%)	
I am in favor of the compulsory vaccines for children approved by the government of Saudi Arabia.	Male	34 (72.3%)	12 (25.5%)	1 (2.1%)	2.814, 0.245
	Female	168 (71.8%)	46 (19.7%)	20 (8.5%)	
Everyone should be able to decide which vaccines are needed for their children.	Male	27 (57.4%)	17 (36.2%)	3 (6.4%)	6.166, 0.046
	Female	167 (71.4%)	46 (19.7%)	21 (9.0%)	
Alternative medicines strengthen the body's defenses, thus leading to a complete cure.	Male	18 (38.3%)	20 (42.6%)	9 (19.1%)	2.158, 0.340
	Female	86 (36.8%)	120 (51.3%)	28 (12.0%)	
I prefer my daughter to develop defenses against papillomavirus infections naturally rather than through vaccination.	Male	13 (27.7%)	24 (51.1%)	10 (21.3%)	2.635, 0.268
	Female	94 (40.2%)	101 (43.2%)	39 (16.7%)	

**Figure 2:**
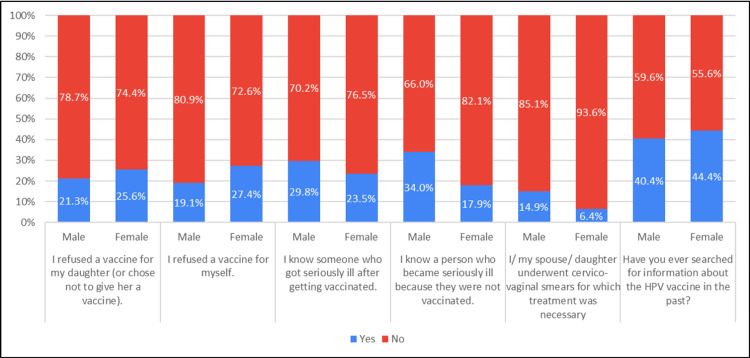
Association of practice with gender (N=281)

The incidence of vaccine refusal for both daughters and themselves exhibited similar patterns across genders. Instances of individuals falling ill after vaccination were comparable between males and females. However, the tendency to abstain from vaccination due to existing illness was notably higher among males. The prevalence of individuals actively seeking information about the HPV vaccine in the past demonstrated similarities between genders. (Table 2, Figure 2).

**Table 2:** Association of practice with gender (N=281)

Practice	Sex	Yes ‘n(%)’	No ‘n(%)’	Chi-square, P-value
I refused a vaccine for my daughter (or chose not to give her a vaccine).	Male	10 (21.3%)	37 (78.7%)	0.399, 0.528
	Female	60 (25.6%)	174 (74.4%)	
I refused a vaccine for myself.	Male	9 (19.1%)	38 (80.9%)	1.369, 0.242
	Female	64 (27.4%)	170 (72.6%)	
I know someone who got seriously ill after getting vaccinated.	Male	14 (29.8%)	33 (70.2%)	.834, 0.361
	Female	55 (23.5%)	179 (76.5%)	
I know a person who became seriously ill because they were not vaccinated.	Male	16 (34.0%)	31 (66.0%)	6.189, 0.013
	Female	42 (17.9%)	192 (82.1%)	
I/ my spouse/ daughter underwent cervico-vaginal smears for which treatment was necessary.	Male	7 (14.9%)	40 (85.1%)	3.903, 0.048
	Female	15 (6.4%)	219 (93.6%)	
Have you ever searched for information about the HPV vaccine in the past?	Male	19 (40.4%)	28 (59.6%)	.257, 0.612
	Female	104 (44.4%)	130 (55.6%)	

**Figure 3:**
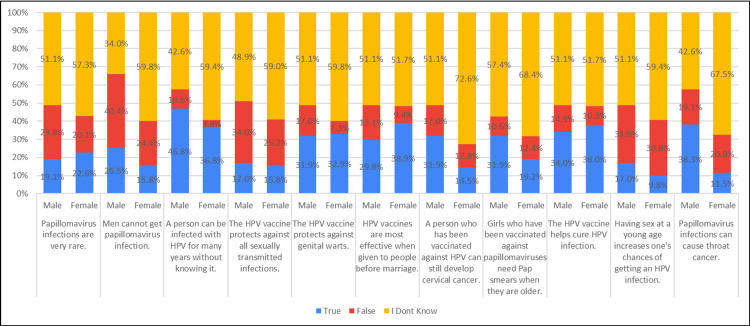
Association of HPV knowledge with gender (N=281)

Approximately 25.5% of males, in contrast to 15.8% of females, held the belief that men cannot contract a papillomavirus infection. About 46.8% of males, compared to 36.8% of females, believed that an individual could be infected with HPV for many years without being aware of it. Around 29.8% of males, as opposed to 38.9% of females, perceived HPV vaccines to be most effective when administered to individuals before marriage. Additionally, approximately 31.9% of males and 14.5% of females believed that individuals vaccinated against HPV could still develop cervical cancer. Lastly, around 38.3% of males and 11.5% of females subscribed to the belief that a papillomavirus infection could lead to throat cancer (Table 3, Figure 3).

**Table 3:** Association of knowledge with gender (N=281)

Knowledge	Sex	True ‘n(%)’	False ‘n(%)’	I Don’t Know ‘n(%)’	Chi-square, P-value
Papillomavirus infections are very rare.	Male	9 (19.1%)	14 (29.8%)	24 (51.1%)	2.182, 0.336
	Female	53 (22.6%)	47 (20.1%)	134 (57.3%)	
Men cannot get papillomavirus infection.	Male	12 (25.5%)	19 (40.4%)	16 (34.0%)	10.544, 0.005
	Female	37 (15.8%)	57 (24.4%)	140 (59.8%)	
A person can be infected with HPV for many years without knowing it.	Male	22 (46.8%)	5 (10.6%)	20 (42.6%)	6.617, 0.037
	Female	86 (36.8%)	9 (3.8%)	139 (59.4%)	
The HPV vaccine protects against all sexually transmitted infections.	Male	8 (17.0%)	16 (34.0%)	23 (48.9%)	1.867, 0.393
	Female	37 (15.8%)	59 (25.2%)	138 (59.0%)	
The HPV vaccine protects against genital warts.	Male	15 (31.9%)	8 (17.0%)	24 (51.1%)	4.714, 0.095
	Female	77 (32.9%)	17 (7.3%)	140 (59.8%)	
HPV vaccines are most effective when given to people before marriage.	Male	14 (29.8%)	9 (19.1%)	24 (51.1%)	4.242, 0.120
	Female	91 (38.9%)	22 (9.4%)	121 (51.7%)	
A person who has been vaccinated against HPV can still develop cervical cancer.	Male	15 (31.9%)	8 (17.0%)	24 (51.1%)	9.936, 0.007
	Female	34 (14.5%)	30 (12.8%)	170 (72.6%)	
Girls who have been vaccinated against papillomaviruses need Pap smears when they are older.	Male	15 (31.9%)	5 (10.6%)	27 (57.4%)	3.751, 0.153
	Female	45 (19.2%)	29 (12.4%)	160 (68.4%)	
The HPV vaccine helps cure HPV infection.	Male	16 (34.0%)	7 (14.9%)	24 (51.1%)	0.933, 0.627
	Female	89 (38.0%)	24 (10.3%)	121 (51.7%)	
Having sex at a young age increases your chances of getting an HPV infection.	Male	8 (17.0%)	15 (31.9%)	24 (51.1%)	2.321, 0.313
	Female	23 (9.8%)	72 (30.8%)	139 (59.4%)	
Papillomavirus infections can cause throat cancer.	Male	18 (38.3%)	9 (19.1%)	20 (42.6%)	21.413, <0.001
	Female	27 (11.5%)	49 (20.9%)	158 (67.5%)	

**Figure 4:**
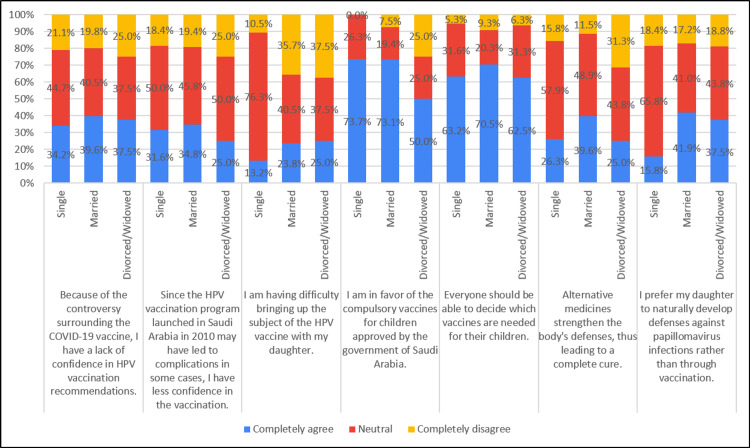
Association of attitude with marital status (N=281)

Approximately 23.8% of married individuals, 25% of divorced individuals, and 13.2% of those with single marital status reported experiencing difficulty discussing the HPV vaccine with their daughters. Views on favoring compulsory vaccines for children approved by the Saudi Arabian government varied, with approximately 73.7% of single individuals, 73.1% of married individuals, and 50% of divorced individuals expressing support. In terms of preferences for their daughters' immunity against papillomavirus infections, around 41.9% of married individuals, 37.5% of divorced individuals, and 15.8% of single individuals favored natural development of immunity over vaccination (Table 4, Figure 4).

**Table 4:** Association of attitudes with marital status (N=281)

Attitude	Marital Status	Completely agree ‘n(%)’	Neutral ‘n(%)’	Completely disagree ‘n(%)’	Chi-square, P-value
Because of the controversy surrounding the COVID-19 vaccine, I have less confidence in HPV vaccination recommendations.	Single	13 (34.2%)	17 (44.7%)	8 (21.1%)	.655, 0.957
	Married	90 (39.6%)	92 (40.5%)	45 (19.8%)	
	Divorced/Widowed	6 (37.5%)	6 (37.5%)	4 (25.0%)	
Since the HPV vaccination program launched in Saudi Arabia in 2010 may have led to complications in some cases, I have less confidence in the vaccination.	Single	12 (31.6%)	19 (50.0%)	7 (18.4%)	.924, 0.921
	Married	79 (34.8%)	104 (45.8%)	44 (19.4%)	
	Divorced/Widowed	4 (25.0%)	8 (50.0%)	4 (25.0%)	
I am having difficulty bringing up the subject of the HPV vaccine with my daughter.	Single	5 (13.2%)	29 (76.3%)	4 (10.5%)	17.636, 0.001
	Married	54 (23.8%)	92 (40.5%)	81 (35.7%)	
	Divorced/Widowed	4 (25.0%)	6 (37.5%)	6 (37.5%)	
I am in favor of the compulsory vaccines for children approved by the government of Saudi Arabia.	Single	28 (73.7%)	10 (26.3%)	0 (0.0%)	11.463, 0.022
	Married	166 (73.1%)	44 (19.4%)	17 (7.5%)	
	Divorced/Widowed	8 (50.0%)	4 (25.0%)	4 (25.0%)	
Everyone should be able to decide which vaccines are needed for their children.	Single	24 (63.2%)	12 (31.6%)	2 (5.3%)	3.517, 0.475
	Married	160 (70.5%)	46 (20.3%)	21 (9.3%)	
	Divorced/Widowed	10 (62.5%)	5 (31.3%)	1 (6.3%)	
Alternative medicines strengthen the body's defenses, thus leading to a complete cure.	Single	10 (26.3%)	22 (57.9%)	6 (15.8%)	7.557, 0.109
	Married	90 (39.6%)	111 (48.9%)	26 (11.5%)	
	Divorced/Widowed	4 (25.0%)	7 (43.8%)	5 (31.3%)	
I prefer for my daughter to develop defenses against papillomavirus infections naturally rather than through vaccination.	Single	6 (15.8%)	25 (65.8%)	7 (18.4%)	10.363, 0.035
	Married	95 (41.9%)	93 (41.0%)	39 (17.2%)	
	Divorced/Widowed	6 (37.5%)	7 (43.8%)	3 (18.8%)	

**Figure 5:**
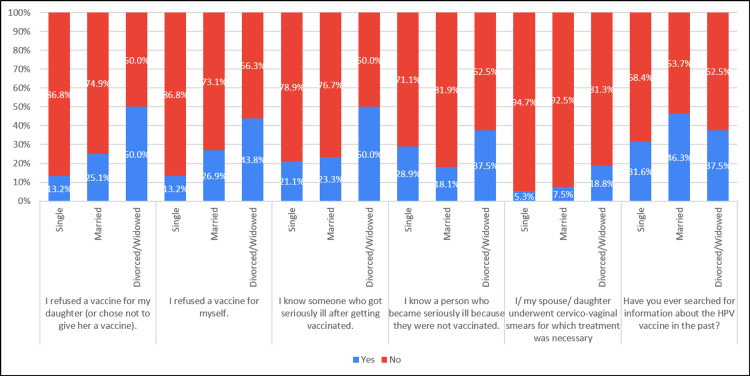
Association of practice with marital status (N=281)

Approximately 13.2% of those with a single marital status, 25.1% of married individuals, and 50% of divorced individuals reported refusing the vaccine for their daughters. Additionally, around 21.1% of those with a single marital status, 23.3% of married individuals, and 50% of divorced individuals were acquainted with someone who experienced serious illness after receiving the vaccine (Table 5, Figure 5).

**Table 5:** Association of practice with marital status (N=281)

Practice	Marital Status	Yes ‘n(%)’	No ‘n(%)’	Chi-square, P-value
I refused a vaccine for my daughter (or chose not to give her a vaccine).	Single	5 (13.2%)	33 (86.8%)	8.195, 0.017
	Married	57 (25.1%)	170 (74.9%)	
	Divorced/Widowed	8 (50.0%)	8 (50.0%)	
I refused a vaccine for myself.	Single	5 (13.2%)	33 (86.8%)	5.970, 0.051
	Married	61 (26.9%)	166 (73.1%)	
	Divorced/Widowed	7 (43.8%)	9 (56.3%)	
I know someone who got seriously ill after getting vaccinated	Single	8 (21.1%)	30 (78.9%)	6.022, 0.049
	Married	53 (23.3%)	174 (76.7%)	
	Divorced/Widowed	8 (50.0%)	8 (50.0%)	
I know a person who got seriously ill because they were not vaccinated.	Single	11 (28.9%)	27 (71.1%)	5.299, 0.071
	Married	41 (18.1%)	186 (81.9%)	
	Divorced/Widowed	6 (37.5%)	10 (62.5%)	
I/ my spouse/ daughter underwent cervico-vaginal smears for which treatment was necessary	Single	2 (5.3%)	36 (94.7%)	3.027, 0.220
	Married	17 (7.5%)	210 (92.5%)	
	Divorced/Widowed	3 (18.8%)	13 (81.3%)	
Have you ever searched for information about the HPV vaccine in the past?	Single	12 (31.6%)	26 (68.4%)	3.120, 0.210
	Married	105 (46.3%)	122 (53.7%)	
	Divorced/Widowed	6 (37.5%)	10 (62.5%)	

**Figure 6:**
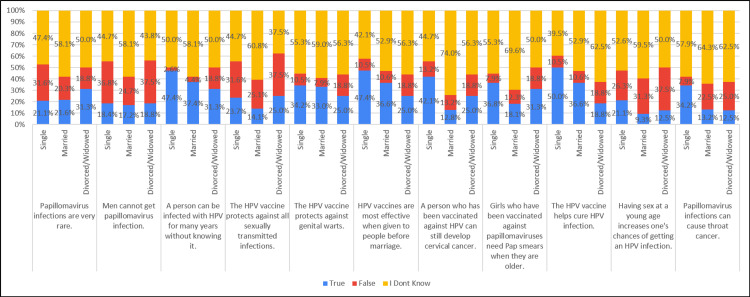
Association of knowledge with marital status (N=281)

Approximately 42.1% of individuals with a single marital status, 12.8% of married individuals, and 25% of divorced individuals believed that a person who has been vaccinated against HPV can still develop cervical cancer. Furthermore, around 34.2% of those with a single marital status, 13.2% of married individuals, and 12.5% of divorced individuals thought that papillomavirus infections could lead to throat cancer (Table 6, Figure 6).

**Table 6:** Association of knowledge with marital status (N=281)

Knowledge	Marital Status	True ‘n(%)’	False ‘n(%)’	I Don’t Know ‘n(%)’	Chi-square, P-value
Papillomavirus infections are very rare.	Single	8 (21.1%)	12 (31.6%)	18 (47.4%)	3.431, 0.488
	Married	49 (21.6%)	46 (20.3%)	132 (58.1%)	
	Divorced/Widowed	5 (31.3%)	3 (18.8%)	8 (50.0%)	
Men cannot get papillomavirus infection.	Single	7 (18.4%)	14 (36.8%)	17 (44.7%)	3.992, 0.407
	Married	39 (17.2%)	56 (24.7%)	132 (58.1%)	
	Divorced/Widowed	3 (18.8%)	6 (37.5%)	7 (43.8%)	
A person can be infected with HPV for many years without knowing it.	Single	18 (47.4%)	1 (2.6%)	19 (50.0%)	8.234, 0.083
	Married	85 (37.4%)	10 (4.4%)	132 (58.1%)	
	Divorced/Widowed	5 (31.3%)	3 (18.8%)	8 (50.0%)	
The HPV vaccine protects against all sexually transmitted infections.	Single	9 (23.7%)	12 (31.6%)	17 (44.7%)	6.602, 0.158
	Married	32 (14.1%)	57 (25.1%)	138 (60.8%)	
	Divorced/Widowed	4 (25.0%)	6 (37.5%)	6 (37.5%)	
The HPV vaccine protects against genital warts.	Single	13 (34.2%)	4 (10.5%)	21 (55.3%)	2.514, 0.642
	Married	75 (33.0%)	18 (7.9%)	134 (59.0%)	
	Divorced/Widowed	4 (25.0%)	3 (18.8%)	9 (56.3%)	
HPV vaccines are most effective when given to people before marriage.	Single	18 (47.4%)	4 (10.5%)	16 (42.1%)	3.429, 0.489
	Married	83 (36.6%)	24 (10.6%)	120 (52.9%)	
	Divorced/Widowed	4 (25.0%)	3 (18.8%)	9 (56.3%)	
A person who has been vaccinated against HPV can still develop cervical cancer.	Single	16 (42.1%)	5 (13.2%)	17 (44.7%)	21.399, 0.000
	Married	29 (12.8%)	30 (13.2%)	168 (74.0%)	
	Divorced/Widowed	4 (25.0%)	3 (18.8%)	9 (56.3%)	
Girls who have been vaccinated against papillomaviruses need Pap smears when they are older.	Single	14 (36.8%)	3 (7.9%)	21 (55.3%)	9.010, 0.061
	Married	41 (18.1%)	28 (12.3%)	158 (69.6%)	
	Divorced/Widowed	5 (31.3%)	3 (18.8%)	8 (50.0%)	
The HPV vaccine helps cure HPV infection.	Single	19 (50.0%)	4 (10.5%)	15 (39.5%)	5.584, 0.232
	Married	83 (36.6%)	24 (10.6%)	120 (52.9%)	
	Divorced/Widowed	3 (18.8%)	3 (18.8%)	10 (62.5%)	
Having sex at a young age increases your chances of getting HPV infection.	Single	8 (21.1%)	10 (26.3%)	20 (52.6%)	5.086, 0.279
	Married	21 (9.3%)	71 (31.3%)	135 (59.5%)	
	Divorced/Widowed	2 (12.5%)	6 (37.5%)	8 (50.0%)	
Papillomavirus infections can cause throat cancer.	Single	13 (34.2%)	3 (7.9%)	22 (57.9%)	12.809, 0.012
	Married	30 (13.2%)	51 (22.5%)	146 (64.3%)	
	Divorced/Widowed	2 (12.5%)	4 (25.0%)	10 (62.5%)	

**Figure 7:**
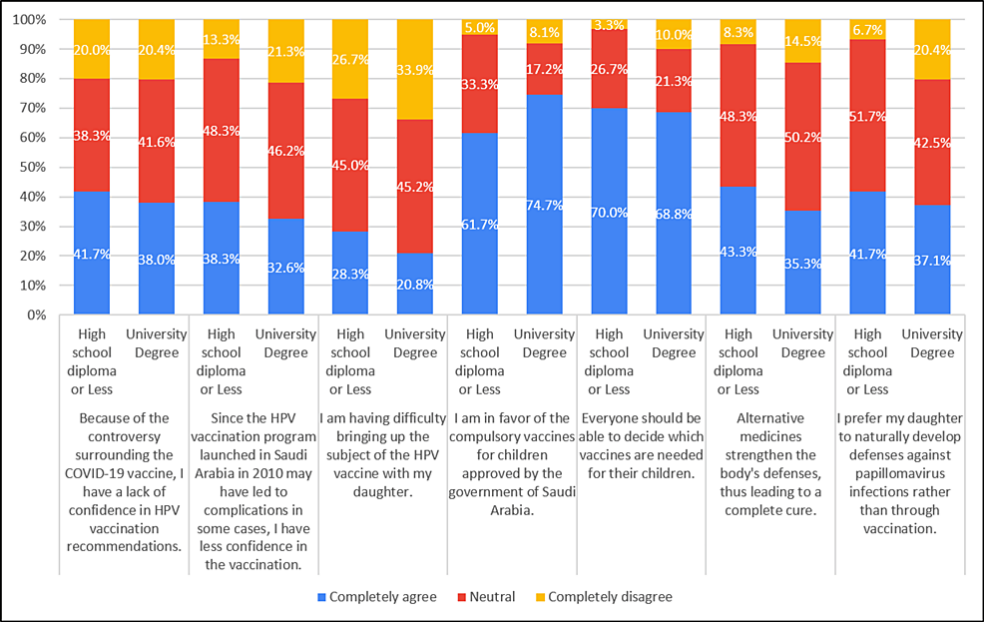
Association of attitude with education level (N=281)

Approximately 61.7% of individuals with a high school diploma or less education, and 74.7% of those with a university degree, expressed support for compulsory vaccines for children approved by the government of Saudi Arabia. In terms of preferences for their daughters' immunity, around 41.7% of individuals with a high school diploma or less education, and 37.1% of those with a university degree, favored natural development of defense against papillomavirus infections over vaccination (Table 7, Figure 7).

**Table 7:** Association of attitudes with education level (N=281)

Attitude	Education	Completely agree ‘n(%)’	Neutral ‘n(%)’	Completely disagree ‘n(%)’	Chi-square, P-value
Because of the controversy surrounding the COVID-19 vaccine, I have less confidence in HPV vaccination recommendations.	High school diploma or Less	25 (41.7%)	23 (38.3%)	12 (20.0%)	.291, 0.865
	University Degree	84 (38.0%)	92 (41.6%)	45 (20.4%)	
Since the HPV vaccination program launched in Saudi Arabia in 2010 may have led to complications in some cases, I have less confidence in the vaccination	High school diploma or Less	23 (38.3%)	29 (48.3%)	8 (13.3%)	2.028, 0.363
	University Degree	72 (32.6%)	102 (46.2%)	47 (21.3%)	
I am having difficulty bringing up the subject of the HPV vaccine with my daughter (skip this question if you do not have a daughter).	High school diploma or Less	17 (28.3%)	27 (45.0%)	16 (26.7%)	1.961, 0.375
	University Degree	46 (20.8%)	100 (45.2%)	75 (33.9%)	
I am in favor of the compulsory vaccines for children approved by the government of Saudi Arabia.	High school diploma or Less	37 (61.7%)	20 (33.3%)	3 (5.0%)	7.687, 0.021
	University Degree	165 (74.7%)	38 (17.2%)	18 (8.1%)	
Everyone should be able to decide which vaccines are needed for their children.	High school diploma or Less	42 (70.0%)	16 (26.7%)	2 (3.3%)	3.046, 0.218
	University Degree	152 (68.8%)	47 (21.3%)	22 (10.0%)	
Alternative medicines strengthen the body's defenses, thus leading to a complete cure.	High school diploma or Less	26 (43.3%)	29 (48.3%)	5 (8.3%)	2.212, 0.331
	University Degree	78 (35.3%)	111 (50.2%)	32 (14.5%)	
I prefer for my daughter to develop defenses against papillomavirus infections naturally rather than through vaccination	High school diploma or Less	25 (41.7%)	31 (51.7%)	4 (6.7%)	6.218, 0.045
	University Degree	82 (37.1%)	94 (42.5%)	45 (20.4%)	

**Figure 8:**
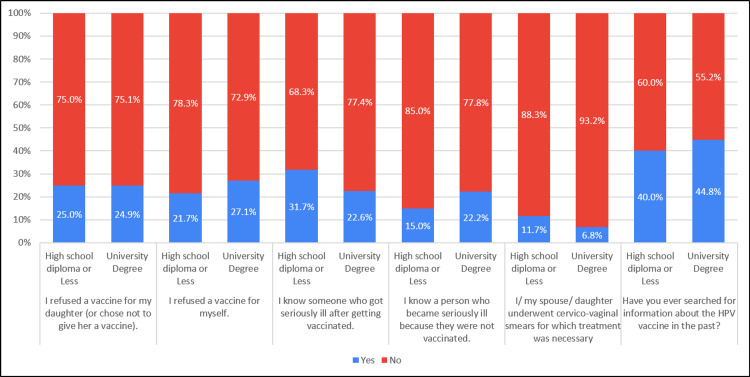
Association of practice with education level (N=281)

The incidence of vaccine refusal for one's daughter, oneself, knowing someone who experienced serious illness after vaccination, being acquainted with a person who became seriously ill due to lack of vaccination, and actively searching for information about the HPV vaccine in the past exhibited similarities between individuals with a high school diploma and those with a university degree (Table 8, Figure 8).

**Table 8:** Association of practice with education level (N=281)

Practice	Education	Yes ‘n(%)’	No ‘n(%)’	Chi-square, P-value
I refused a vaccine for my daughter (or chose not to give her a vaccine).	High school diploma or Less	15 (25.0%)	45 (75.0%)	.000, 0.986
	University Degree	55 (24.9%)	166 (75.1%)	
I refuse a vaccine for myself.	High school diploma or Less	13 (21.7%)	47 (78.3%)	.738, 0.390
	University Degree	60 (27.1%)	161 (72.9%)	
I know someone who got seriously ill after getting vaccinated.	High school diploma or Less	19 (31.7%)	41 (68.3%)	2.083, 0.149
	University Degree	50 (22.6%)	171 (77.4%)	
I know a person who got seriously ill because they were not vaccinated.	High school diploma or Less	9 (15.0%)	51 (85.0%)	1.482, 0.223
	University Degree	49 (22.2%)	172 (77.8%)	
I/ my spouse/ daughter underwent cervico-vaginal smears for which treatment was necessary	High school diploma or Less	7 (11.7%)	53 (88.3%)	1.557, 0.212
	University Degree	15 (6.8%)	206 (93.2%)	
Have you ever searched for information about the HPV vaccine in the past?	High school diploma or Less	24 (40.0%)	36 (60.0%)	.441, 0.507
	University Degree	99 (44.8%)	122 (55.2%)	

**Figure 9:**
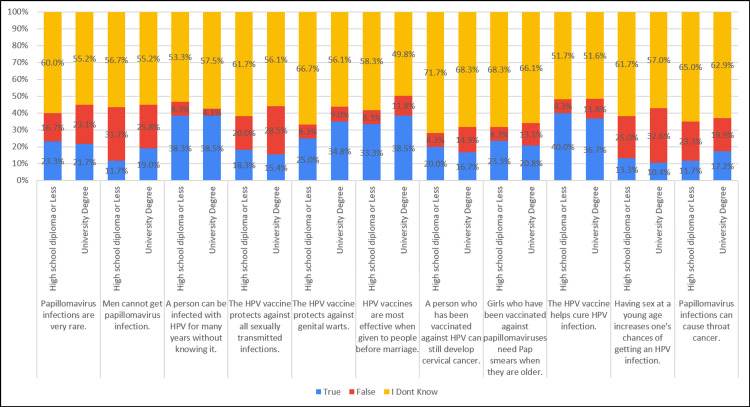
Association of knowledge with education level (N=281)

The perspectives on men being unable to contract papillomavirus infection, the possibility of HPV infection for an extended period without awareness, the perceived efficacy of HPV vaccines when administered before marriage, the belief that individuals vaccinated against HPV could still develop cervical cancer, and the belief in the potential of papillomavirus infection to cause throat cancer were similar between individuals with a high school diploma and those with a university degree (Table 9, Figure 9).

**Table 9:** Association of knowledge with education level (N=281)

Knowledge	Education	True ‘n(%)’	False ‘n(%)’	I Don’t Know ‘n(%)’	Chi-square, P-value
Papillomavirus infections are very rare.	High school diploma or Less	14 (23.3%)	10 (16.7%)	36 (60.0%)	1.142, 0.565
	University Degree	48 (21.7%)	51 (23.1%)	122 (55.2%)	
Men cannot get papillomavirus infection	High school diploma or Less	7 (11.7%)	19 (31.7%)	34 (56.7%)	2.077, 0.354
	University Degree	42 (19.0%)	57 (25.8%)	122 (55.2%)	
A person can be infected with HPV for many years without knowing it.	High school diploma or Less	23 (38.3%)	5 (8.3%)	32 (53.3%)	1.862, 0.394
	University Degree	85 (38.5%)	9 (4.1%)	127 (57.5%)	
The HPV vaccine protects against all sexually transmitted infections.	High school diploma or Less	11 (18.3%)	12 (20.0%)	37 (61.7%)	1.790, 0.409
	University Degree	34 (15.4%)	63 (28.5%)	124 (56.1%)	
The HPV vaccine protects against genital warts.	High school diploma or Less	15 (25.0%)	5 (8.3%)	40 (66.7%)	2.325, 0.313
	University Degree	77 (34.8%)	20 (9.0%)	124 (56.1%)	
HPV vaccines are most effective when given to people before marriage.	High school diploma or Less	20 (33.3%)	5 (8.3%)	35 (58.3%)	1.506, 0.471
	University Degree	85 (38.5%)	26 (11.8%)	110 (49.8%)	
A person who has been vaccinated against HPV can still develop cervical cancer.	High school diploma or Less	12 (20.0%)	5 (8.3%)	43 (71.7%)	1.883, 0.390
	University Degree	37 (16.7%)	33 (14.9%)	151 (68.3%)	
Girls who have been vaccinated against papillomaviruses need Pap smears when they are older.	High school diploma or Less	14 (23.3%)	5 (8.3%)	41 (68.3%)	1.071, 0.585
	University Degree	46 (20.8%)	29 (13.1%)	146 (66.1%)	
The HPV vaccine helps cure HPV infection.	High school diploma or Less	24 (40.0%)	5 (8.3%)	31 (51.7%)	.645, 0.724
	University Degree	81 (36.7%)	26 (11.8%)	114 (51.6%)	
Having sex at a young age increases your chances of getting HPV infection.	High school diploma or Less	8 (13.3%)	15 (25.0%)	37 (61.7%)	1.418, 0.492
	University Degree	23 (10.4%)	72 (32.6%)	126 (57.0%)	
Papillomavirus infections can cause throat cancer.	High school diploma or Less	7 (11.7%)	14 (23.3%)	39 (65.0%)	1.201, 0.548
	University Degree	38 (17.2%)	44 (19.9%)	139 (62.9%)	

## Discussion

The primary aim of this research is to assess the level of knowledge and awareness among parents regarding HPV, including its associated health risks and the benefits of vaccination. Almost 281 individuals participated in this study. Of these, 17% were men, while 83% were women.

Our current study found notable gender disparities in attitudes and beliefs regarding vaccination, particularly concerning the COVID-19 and HPV vaccines. Females generally expressed higher levels of agreement with vaccination recommendations and demonstrated greater confidence in the safety and efficacy of vaccines compared to their male counterparts. This discrepancy extended to various aspects, including the perceived controversy surrounding the COVID-19 vaccine, willingness to discuss the HPV vaccine, and support for routine vaccines for children. These findings are consistent with those of a previous study which indicated a prevalent lack of understanding about HPV infection and vaccination within the targeted population [11, 12]. In contrast, only a limited number of studies, often with small sample sizes, have been conducted to explore the knowledge of Saudis regarding cervical cancer and the uptake of HPV vaccination [13]. In comparison to similar studies, one conducted among 181 Saudi medical students at King Faisal University in 2014 revealed that the majority of participants had limited awareness of early warning signs, symptoms, and risk factors associated with cervical cancer. The accuracy of responses ranged from 43.7% to 55% on average [14]. Similarly, a study assessing the overall knowledge of cervical cancer among students aged 17 to 26 in Poland found that knowledge was insufficient, with HPV infection not being recognized as the primary cause [15]. Notably, a recent survey among healthcare practitioners in Greece exposed a substantial knowledge gap concerning HPV, as only 30% of the sample appeared to be aware of its significant role in carcinogenesis. A recent poll in Thailand also indicated a low level of understanding regarding HPV [16].

Approximately one-third of the participants in the current study (32.9%) possessed knowledge about the HPV vaccine, with 19.2% perceiving the vaccine to have various side effects. Notably, physicians were the primary source of information for the majority (38.0%) of these participants. These findings align with a prior study conducted in Saudi Arabia in 2014, revealing that 67% of participants were unaware of the existence of HPV vaccination [14]. Many women have little information about HPV and cervical cancer, and a number of factors, including cost, safety, efficacy, and knowledge of the vaccine, influence women's adoption of HPV vaccination globally [17].

A recent study conducted in Saudi Arabia in 2023 found that levels of vaccine acceptance were high (54.7%) although (41.0%) had a negative attitude towards the effectiveness of the HPV vaccine with a strong belief that the vaccine can cause serious side effects (67.0%). Parents with a family history of cervical cancer and were aware that the vaccine can prevent cervical cancer were positively associated [18].

Further, the current study highlighted significant variations in viewpoints based on educational levels. Participants with higher education levels, i.e., university degree graduates, tended to demonstrate stronger support for compulsory vaccines for children and were more likely to prefer the natural development of immunity against HPV infections for their daughters. These findings suggest that education plays a crucial role in shaping individuals' attitudes towards vaccination. Moreover, the study uncovered disparities in beliefs related to HPV infection and its consequences. Notably, a considerable percentage of participants, particularly males, held misconceptions about the risks and outcomes of HPV vaccination, such as the belief that vaccinated individuals could still develop cervical cancer or that a papillomavirus infection could lead to throat cancer. These misconceptions highlight the importance of targeted educational interventions to improve understanding and dispel myths surrounding HPV and vaccination. A similar study conducted in Sharjah, United Arab Emirates illustrated a significant correlation was found between the spouse’s level of education, HPV (Pearson-chi square value: 5.049 and p: 0.025), and HPV vaccine (Pearson-chi square value: 4.057 and p:0.044) [19]. Another study indicated a noteworthy association between the level of education and awareness of the male spouse regarding HPV and its vaccine [20]. This shows that husbands have the ability to impact their wives' or daughters' decisions regarding vaccination uptake. If newly developed immunization programs addressed both men and women, better immunization outcomes may be anticipated. The results of a recent cross-sectional study carried out in the Emirate of Abu-Dhabi are comparable to these [20]. 

Limitations of the study

The limitation of the study was the small sample size used. This decreased the ability to generalize the results. Furthermore, given that parents completed the questionnaires themselves, there may have been response bias in the study as well, leading them to provide answers that were socially acceptable.

## Conclusions

The study highlights significant disparities in knowledge, attitudes, and beliefs related to vaccines, particularly the HPV vaccine, among the surveyed population. The findings suggest a pervasive lack of understanding regarding HPV, its associated risks, and the benefits of vaccination. Notably, gender, education level, and marital status emerged as influential factors shaping these perspectives. Furthermore, the study draws attention to the limited awareness and understanding of the HPV vaccine, with a substantial portion of participants being uninformed or harboring misconceptions. These findings underscore the necessity for comprehensive public health initiatives to enhance awareness, correct misinformation, and promote vaccine uptake. Overall, the study contributes to our understanding of the factors influencing vaccine-related perceptions in the surveyed population. The identified disparities call for multifaceted interventions, including educational programs, targeted awareness campaigns, and policy considerations, to address the nuanced challenges surrounding HPV vaccination and improve overall vaccine literacy within the community.
